# An Overview of the Strengths and Challenges Related to Health on the First 10 Days after the Large Earthquake in the West of Iran, 2017

**Published:** 2019-05

**Authors:** Mahmoudreza PEYRAVI, Milad AHMADI MARZALEH, Amir KHORRAM-MANESH

**Affiliations:** 1. Department of Health in Disasters and Emergencies, Health Human Resources Research Center, School of Management and Medical Informatics, Shiraz University of Medical Sciences, Shiraz, Iran; 2. Student Research Committee, School of Management and Medical Informatics, Shiraz University of Medical Sciences, Shiraz, Iran; 3. Department of Surgery, Institute of Clinical Sciences, Sahlgrenska Academy, University of Gothenburg, Gothenburg, Sweden

**Keywords:** Earthquake, Healthcare, Disaster management, Iran

## Abstract

**Background:**

The objective of the present study was to investigate the strength and weaknesses of healthcare management during the first 10 days after the earthquake in Sarpol-e Zahab in Kermanshah, Iran.

**Methods:**

This qualitative, observational study was conducted on November 13–23, 2017 in the disaster area, by using content analysis. Data was collected through experts and focus group interviews with professional and healthcare staff, and policy-makers.

**Results:**

Our findings were categorized into 7 major groups; environmental health; mental health; mothers, infants and children’s health; field hospital; nutrition; contagious diseases; drug delivery. There were good cooperation and coordination regarding environmental health issues. However, other categories were handled by different organizations and resulted in a chaotic situation.

**Conclusion:**

The post-earthquake period is overwhelmed with considerable issues regarding the care of victims and therapeutic measures. Lack of quick, reliable, and appropriate management will result in extensive health issues, including epidemic, worsening of chronic diseases, and exacerbation of mental disorders.

## Introduction

Iran is one of the 10 disaster-prone countries in the world with 90% of its population exposed to earthquake and flooding. Statistically speaking, Iran has been ranked six in terms of the incident of natural events (1, 2). Several fatal earthquakes have occurred in Iran so far, among which the earthquakes in Bam and Rudbar had a higher rate of deaths (3). Different studies have discussed the post-earthquakes problems with regard to healthcare efficiency (4–7) since without the timely and appropriate medical actions, the health and lives of the people can be threatened with no possibility to prevent high mortality and morbidity.

Ezgeleh is a city in Kermanshah Province, located in Ezgeleh district, Salas-e Babajani County, with a population of 1502 people according to 2016 census of population (8), and an area of 900 m2. An earthquake with the magnitude of 7.3 hit the city on November 12, 2017, at 9:48 P.M. The depth of the earthquake was 11 kilometers and its focus in Ezgeleh, which was the closest city to the seismic focus (with a 5-kilometer distance). Due to the vicinity to Sarpol-e Zahab city, Ezgeleh was severely damaged. Based on the last statistics of the legal medical authorities in Iran, of around 620 deaths in Kermanshah earthquake, 559 died in Sarpol-e Zahab. Approximately, 9000 people were also injured and 70000 people became homeless (9, 10). The innovation of this study is that the experts who were helping the people were interviewed at the same time. Also, a study on the strengths and challenges of the earthquake in Kermanshah has not yet been published.

Regarding the importance of the quick and vital responsiveness of the health system, especially the human force and facilities related to health, this study was done and aimed at investigating the strengths, weaknesses, and the health-related needs in the first 10 days after the large earthquake in the west of Iran by interviewing the experts in 2017; it is hoped that the results of the present study would be useful for the probable disasters in the future.

## Methods

### Study design

This qualitative, observational study was conducted on November 13–23, 2017 in regions affected by earthquake including Sarpol-e Zahab, Ezgeleh, Eslamabad-e Gharb, Salas-e Babajani, Kerend-e Gharb, and Qasr-e Shirin in Kermanshah Province. This study was conducted in Sarpol-e Zahab, which is an endemic region of cutaneous leishmaniosis.

### Data collection

Data were collected through observation, and in-depth interview with 54 experts and stakeholders, and five focus groups consisting of experts, employees, and policymakers. Since the researcher was an expert of health in disasters, a part of data collection was done with observation. All observations were registered and interviews and meetings were recorded and were analyzed by authors.

### Participants

Inclusions criteria for participants and focus groups; 1) being actively involved in the disaster area, and 2) Working experience of at least for 2 years. Exclusions criteria for participants and focus groups; 1) reluctance to participate in the study.

### Measurement

A total number of 54 experts participated in the semi-structured and in-depth interviews. Semi-structured questions about the strengths and challenges of the health system services were asked from the experts. Probing questions were also asked based on the answers of the participants. The interviews lasted approximately 20–40 minutes. They were conducted in a quiet place. After each interview, it was immediately transcribed and the weaknesses, strengths, and challenges were extracted.

Finally, 5 focus group sessions were held; the number of the people in each of the focus groups varied from 7 to 10 people. The duration of each focus group interview was between 1–2 hours. The sessions were directed and coordinated by the researcher. Semi-structured questions were asked from each expert in their profession regarding the undertaken actions, and measures, strengths, weaknesses, challenges, and needs. Each point was discussed in detail if needed. Data were analyzed, using the Inductive and Conventional approach based on the Graneheim and Lundman method.

### Ethics Consideration

All participants in the study completed the informed consent form, and they could withdraw from the study at any stage of the study. The text of the interview was also completely confidential and the participants’ demographics remained confidential.

## Results

The research findings were categorized into 7 major groups of environmental health, mental health, mothers’, infants’, and children’s health, field hospital, nutrition, contagious diseases, and drug. The strengths and challenges of each issue are presented below.

Table 1 presents the number and demographic information of the experts who were interviewed. Thematic table is shown in Table 2.

**Table 1:** The experts in the research

***Row***	***Expert***	***No.***	***Average age (yr)***	***Gender***		***Educational level***				
				***Male***	***Female***	***B.S***	***M.S***	***Ph.D.***	***M.D***	***Fellow and Attend***
1	Environmental health	10	37.2	8	2	10	0	0	0	0
2	Psychologist	10	31.32	5	5	7	3	0	0	0
3	Nutrition	2	34.5	0	2	2	0	0	0	0
4	Emergency medicine	5	36.3	4	1	0	0	0	0	5
5	Pediatrics	1	38	0	1	0	0	0	0	1
6	Radiologist	1	43	1	0	1	0	0	0	0
7	Medical laboratory	1	42	1	0	1	0	0	0	0
8	General practitioner	10	29.3	4	6	0	0	0	10	0
9	Contagious diseases	6	38.8	6	0	6	0	0	0	0
10	Health in disasters and emergencies	2	37.3	2	0	0	0	2	0	0
11	Midwife	3	32.2	0	3	1	1	0	0	0
12	Pharmacist	3	36.3	3	0	0	0	0	3	0
	Total	54	36.35	34	20	28	4	2	13	6

**Table 2:** Thematic table

***Category***	***Sub-Category***	***Sub-Sub-Category***
Environment Health	Challenges	lack of trash bags
		Inappropriate burial of garbage
		Inappropriate transportation and distribution of the hot food
		Disposal of animal manure in rural environments
		The presence of louse and scabies
	Strengths	Visiting the temporary housing camps
		Visiting food preparation and distribution centers
		Disinfection
		Control the rodents
		Wastewater management
Mental Health	Challenges	Overexcitement
		Sleep disorder
		Insomnia
		Suicidal tendency
		Lack of psychologists
	Strengths	The psychologists screened all of the people
		Primary training
Mothers’, infants’, and children’s health	Challenges	Lack of contraceptives
		Lack of fetal electrocardiography
		Lack of multivitamins
		Lack of midwifery instrument
		Non-sterilization of the tools
		Lack of midwives and gynecologists
	Strengths	Visiting and examining the pregnant women and the children
		Examining the nutritional status of the infants
		Conducting delivery
		Visiting the girls and women in reproductive age
		Distributing baby formulas
		Checking the vital signs
Field hospital	Challenges	Lack of drugs
		Lack of surgical instruments
		Lack of staff and specialist
		Few number of security forces
	Strengths	Various medical specialties
Nutrition	Challenges	Lack of nutrition specialists
		The distribution of the expired foods
		Lack of coordination between the organization
	Strengths	Quick assessment of the children
		Screening the nutritional status
		Training proper nutrition to the patients
		Distributing baby formulas and supplements
Contagious diseases	Challenges	Outbreak of infectious diseases
		The syndromic care system was established
	Strengths	Pediatric vaccination was carried out
Drug	Challenges	Shortage of the general drugs
		Lack of control over the distribution of drugs
	Strengths	The drugs were distributed in especial center
		There were vital medicines available

### Environmental health

Strengths: very appropriate measures had been adopted regarding the control and inspection of the drinking water resources, providing healthy water by adding Chlorine, turbidity test, monitoring the mobile water tanks and Chlorine residual testing, visiting the temporary housing camps, monitoring the kitchens, visiting food preparation and distribution centers, and disinfection of the mobile toilets and baths by Chlorine. In the first 8 days after the earthquake, the Chlorine residual in the water was 0 ppm and the turbidity of the urban water was over 5 NTU; however, the Chlorine residual level gradually rose to over 1.5 ppm and turbidity of the water fell to lower than 5 NTU.

Challenges: various problems were observed such as lack of human force and the necessary equipment for the environmental health teams, lack of trash bags in the tents of the earthquake survivors, inappropriate burial of garbage, release of leachate in the environment because of raining, inappropriate transportation and distribution of the hot food, distribution of moldy bread and rancid dates among the affected people, disposal of animal manure in rural environments, presence of the corpses of the cattle and livestock in the city, lack or a few number of healthy water tanks and restrooms in the city and villages, and the presence of louse and scabies.

Lack of restrooms and weak disposal of wastes and wastewater are two serious dangers which increase the incidence probability of contagious diseases among the earthquake survivors.

### Mental health

Strengths: in the affected region, the psychologists screened all of the people and referred the individuals with the acute disorder to the psychiatrist. The actions taken by the mental health team included establishing an effective communication with families, sympathy and expression of feelings in order to gain confidence, and identify primary psychological reactions, and signs and symptoms of anxiety.

Challenges: the majority of the disorders reported in the affected region included anxiety, the stress of the reoccurrence of the incident, overexcitement, sleep disorder and insomnia, suicidal tendency, and complaint from religious principles. Furthermore, there was no necessary equipment including whiteboard, marker, color pencil, wax oil crayon, toy, painting notebook, and gift for children which are necessary for the mental health team.

One of the experts said: “*I’ve been addressing the people, and I realized that a lot of people were afraid and anxious. And the complaints of religious rituals were much higher*”.

### Mothers’, infants’, and children’s health

Strengths: some necessary measures were taken for the affected society including visiting and examining the pregnant women and the children below one year old, examining the nutritional status of the infants with breastmilk and needs assessment of the nutrition of children and pregnant women, conducting delivery, visiting the girls and women in reproductive age, providing services for seniors and the children below one year old, helping vulnerable groups, distributing baby formulas, diapers, sanitary pads, baby bottles, distributing the facilities for birth control among people, vaginal examination (if needed), and checking the vital signs and fetal heartbeat.

Challenges: lack of contraceptives, especially condoms, lack of fetal electrocardiography, lack of multivitamins and, in some cases, excessive intake of vitamins, lack of midwifery Sonic aids and Pinard, thermometer, otoscope, ophthalmoscopy, flashlight, tongue depressor, gloves and mask, blood pressure measurement device, baby scale, suture thread, and dressing set were seriously felt. Moreover, there were very few drugs such as methyldopa pill, magnesium sulfate and hydralazine ampoule for the pregnant women affected by high blood pressure.

### Field hospital

Strengths: various medical specialties were accessible in the mobile hospital.

Challenges: some field hospitals were located on the outskirts of the city and the considerable point was the very low distance of the 3 field hospitals from each other. The assessment of the location of the field hospitals was very incomplete. There were problems such as imperfect setup of Incident Command System (ICS), absence of the environmental health personnel for spraying the hospital, lack of drugs, surgical instruments, and operating room, lack of the emergency box and surgical sets, lack of a helicopter near the hospital to dispatch sick patients, lack of specialties of orthopedics, internal medicine, emergency medicine, pediatricians, general practitioners and nurses (especially female nurses), lack of radiological devices, and few number of security forces in the field hospitals.

### Nutrition

Strengths: in the first week, the following works were done: quick assessment of the children below 5 years old using mid-upper arm circumference, assessment of the malnutrition status of the children below 5 years old, screening the nutritional status of the infants and nursing mothers, providing proper nutrition to the patients with diabetes and those with high blood pressure, investigating the diet portion of the families, A+D supplements and calcium for the children below 2 years old, iron pill, and multivitamins.

Challenges: lack of nutrition specialists was seriously felt in the first 10 days after the earthquake. In the first week after the earthquake, hot food was distributed among the earthquake survivors; there were some problems regarding the health status of the food and its maintenance. The distribution of the expired tuna and canned beans exacerbated the nutritional condition of the people. Lack of coordination between the Ministry of Health and Medical Education and the Red Crescent Society was also seriously felt regarding the food supply and distribution.

### Contagious diseases

Strengths: the syndromic care system was established by the employees of the contagious diseases. No case of contagious diseases was discovered in the first 10 days. Pediatric vaccination was carried out based on the immunization program; however, diphtheria and tetanus vaccines were injected in some cases. Fortunately, no case of Cholera, Measles, and Meningitis was observed in the affected region.

Challenges: the specialists diagnosed some cases of Pediculosis and Scabies. Due to the cold weather, many people caught a cold. There were many cases of acute bloody diarrhea, especially in the children. Regarding the fact that the affected region is an endemic region of Cutaneous leishmaniosis, there should be an appropriate control over the reduction of the Phlebotomus.

One expert said: “Given that the earthquake has damaged urban and rural water pipes, immediately” the pipes should be replaced or rebuilt to prevent the spread of contagious diseases”.

### Drug

Strengths: most of the drugs were given to those who needed them. The drugs were distributed in especial centers such as health centers, rural health centers, and mobile hospitals.

Challenges: There was a severe shortage of these drugs; anti-cough and allergy syrups of Diphenhydramine, Dextromethorphan, and Ketotifen; antibiotics of Amoxicillin, Cefixime, and Azithromycin; the ophthalmic drugs of Sulfacetamide and Chloramphenicol; gastrointestinal pills and painkillers such as gelofen, acetaminophen and Codeine-acetaminophen; Piroxicam gel; and drugs for reducing blood pressure (Captopril) and diabetes (Metformin and Glibenclamide); there was a severe shortage of these drugs. Due to high levels of stress among the earthquake survivors, many of them were affected by Herpes simplex; hence, Aciclovir ointment was consumed to a large extent. There was a shortage of the general drugs such as alcohol pad, sieve, Betadine, the drugs such as gelofen, Chloramphenicol, Betamethasone, Piroxicam gel and A+D, eye drops, and painkillers. The drugs were distributed based on the words of the patients themselves and there was no examination regarding the necessity of the drug by a pharmacist or pharmacist technician; in fact, there was no monitoring.

One of the experts said: “Health care staff distributed widely and widely drugs among people, which could create long-term problems for the people in the future. There is no supervision on the distribution of drugs at all”.

## Discussion

Natural disasters mostly occur in the developing countries due to the lack of a comprehensive emergency response program (11, 12). The health-related needs are manifested more in the initial phase of the disasters (12). In the temporary phase of disasters, medical measures and prevention can prevent the incident of epidemics of contagious diseases (13–15). In the areas affected by the earthquake, the health-related shortages and challenges are more than the medical Strengths.

The study of Udomratn (2008) showed that, after the occurrence of the natural disasters, the prevalence of psychological disorders and social functions, especially the Post-traumatic stress disorder (PTSD) in the earthquake survivors is between 8.6% and 57.3% in terms of intensity, kind of disaster, and individual characteristics (16). Other studies have revealed that psychological symptoms may appear for months, even years after the disaster (17, 18). Consequently, psychological measures need to be carried out with more accuracy and attention to prevent long-term and severe psychological impacts among the affected population.

The incident of infectious diseases increases after the earthquake due to injuries, increased wastes, and the poor environmental health status of the affected region (19). Respiratory diseases were prevalent after the earthquake of Haiti and that of Sichuan (20, 21). Consequently, the population at risk should be identified as soon as possible to prevent epidemics and infectious outbreak.

Few studies have been conducted on the impacts of the earthquake on the pregnant women (22, 23). Because of the earthquake, the pregnant women’s stress results in a reduced weight of the baby (23), disorders during delivery (6), preterm delivery (22, 24), and low growth of the baby head circumference (22, 23). The results of this study, in comparison to others, demonstrate a need for the presence of midwives among the crisis teams’ members since women and children constitute the most vulnerable population of the society. A midwife can be beside the women and give them solace and confidence so as to overcome the difficult conditions of the earthquake.

In the great east Japan earthquake, there was lack of soluble drugs such as glucose 50%, oxytocin, phtharal, olopatadine tablets, normal saline, and levothyroxine tablet (25). The results of this study compared with other studies showed that, regarding the pharmaceutical shortage in Sarpol-e Zahab, it can be said that most of the shortages in Japan are related to special drugs while there was mostly lack of general drugs in Sarpol-e Zahab.

Many health-related services were provided in Bam earthquake (3), which were very similar to those provided in Kermanshah earthquake. This issue indicates that after 15 years of Bam earthquake (3), some problems and inefficiencies are still being repeated in Kermanshah earthquake; hence, there is a need for a new approach to lessons learned, better collaboration and coordination to desirably respond to disasters.

The Iranian healthcare system, including hospitals, prehospital entities, healthcare centers, and other involved stakeholders need a coordinated readiness plan to cope with new incidents and crises (26). This need was seriously felt in the region affected by the earthquake, since there were many cases of inconsistencies and parallelism among the emergency organizations that resulted in the lack of appropriate and quick responsiveness to the earthquake.

### Recommendations

Since Iran is prone to disasters, the occurrence of earthquakes is inevitable. Therefore, it is necessary to plan and implement appropriate measures for higher readiness and mitigation ability. Regional disaster plans for rapid healthcare response should be written, reviewed and revised at least once or twice a year. Furthermore, these plans should be tested by holding table top and operational exercises to identify new risks and weaknesses. Elimination of risks and mitigating disasters consequences enable a quick return to normalcy after a disaster such as an earthquake.

There are some limitations to this study. One of the limitations of the study was the lack of access to all key informants for interviewing. Another limitation is the fact that the results reflect the observation by one researcher, whose impression and experience may have had an impact on the comprehension of the results.

## Conclusion

There were many deficiencies in healthcare response to Kermanshah earthquake, of which the most important one was the intra- and extra-organizational inconsistency and lack of human force and equipment, which resulted in more chaos in the affected region. Since Sarpol-e Zahab is an endemic region of cutaneous leishmaniosis with considerable health issues, rapid and appropriate measures should be implemented to prevent extensive health issues, including epidemics of contagious diseases, worsening of chronic diseases, and the exacerbation of mental disorders. Improved collaboration and coordination among different agencies are a necessary step, which may only be possible by a united regional leadership. Resource management, information-sharing, and training are all natural steps towards an increased healthcare readiness to combat disasters and to provide better services to the victims.

## Ethical considerations

Ethical issues (Including plagiarism, informed consent, misconduct, data fabrication and/or falsification, double publication and/or submission, redundancy, etc.) have been completely observed by the authors.
